# ARMED to ESCAPE COVID-19: the impact of COVID-19 on a mixed methods feasibility study of a weight management, education and physical function programme for patients with knee osteoarthritis at the primary/secondary care interface

**DOI:** 10.1017/S1463423621000359

**Published:** 2021-06-24

**Authors:** Brenda Monaghan, Lara Bourton Cassidy, Aaron A. Glynn, M.V. Hurley, Pauline Kacprzak, Maria Kelly, Keelan O’ Connor, Jane O. Flynn, Fiona Keelan, Ruth Reidy, Sarah Jane Rodgers, Tara Cusack

**Affiliations:** 1 Discipline of Physiotherapy, School of Medicine, Trinity Centre for Health Sciences, Trinity College Dublin, Ireland; 2 Physiotherapy Department, Our Lady’s Hospital, Navan, Ireland; 3 Department of Orthopaedic Surgery, Our Lady’s Hospital, Navan, Ireland; 4 Department of Applied Health and Social Care Research, St George’s University of London and Kingston University, London, UK; 5 Physiotherapy Department, Meath Primary Care, Child and Family Centre, Navan, Co Meath Dublin, Ireland; 6 Health Innovation Hub Ireland (HIHI), Munster Technological University, Cork, Ireland; 7 Department of Nursing, Our Lady’s Hospital, Navan, Ireland; 8 Clinical Nutrition Consultancy, CORU Registered Dietitian and Member of Irish Nutrition and Dietetic Institute, Cork, Ireland; 9 Department of Dietetics, Our Lady’s Hospital, Navan, Ireland; 10 UCD School of Public Health Physiotherapy and Sports Science, Health Sciences Centre, Belfield, Dublin, Ireland

**Keywords:** interface care, knee osteoarthritis, multidisciplinary teams, qualitative research, weight management

## Abstract

**Objectives:**

This study was designed to test the feasibility of running a trial to compare the effectiveness of a combined weight management and physical function programme for patients with knee osteoarthritis *ARMED* (Arthritis Rehabilitation through the Management of Exercise and Diet) with usual care *ESCAPE pain* (Enabling Self-management and Coping with Arthritic Pain using Exercise). The COVID-19 pandemic interruption allowed additional measurement of the qualitative ‘lived in’ experiences of this patient group during the pandemic and also their appetite for virtual health.

**Participants:**

Thirty-two patients with knee osteoarthritis were recruited from a combined primary/secondary care waiting list and were allocated to either a six-week intervention group (ARMED) or to the six-week usual care ESCAPE pain group (Enabling Self-management and Coping with Arthritic Pain using Exercise) group.

**Results:**

The intervention programme was interrupted after three weeks by COVID-19. Fifteen patients were reassessed after the first stage. The average attendance was 92% with 6 patients attending all sessions, 5 attending 5/6, 1 attending 4/6 and 2 attending 3/6. One subject dropped out and 15/16 patients completed all outcome measurements. All patients completed the KOOS knee score and the Short Warwick-Edinburgh Mental Well Being Scale to evaluate anxiety and depression. There was a statistically significant improvement in pain, activities of daily living, quality of life and mental health and well-being scores from time one to time 2. The mean weight, BMI and waist measurements were reduced also from time one to time 2, but these failed to reach significance. The semi-structured interviews provided rich information on enablers and barriers to coping in lockdown, benefits of the ARMED programme to increasing physical activity and weight management and enablers and barriers to redesigning the programme for online delivery.

**Conclusions:**

Evaluation of preliminary data from this feasibility study supports the three-week intervention combining education, exercise and weight management in this patient group even during a pandemic. Based on the results of the qualitative interviews, we have now redesigned our programme to present it virtually. We hope to present the results of our virtual feasibility study later in 2021.

## Introduction

Knee osteoarthritis (O.A) affects 400 000 people in Ireland with 2,206 converting annually to total knee replacement (HIQUA 2014). Although best practice guidelines (NICE 2020) advocate the combination of information, exercise and weight management as the first line of treatment in knee (O.A), there no evidence to our knowledge of this combined intervention in the Irish health service.

In September 2019, we sought and received Slainte Care Integration funding to design and run a feasibility study to compare the effect of a combined intervention ARMED (Arthritis Rehabilitation through the Management of Exercise and Diet) with usual care ESCAPE pain (Enabling Self-management and Coping with Arthritic Pain using Exercise programme) on weight, pain and physical function in patients with knee osteoarthritis at the primary/secondary interface. This feasibility project ARMED commenced in February 2020 as an interface primary/secondary care project.

Unfortunately, the programme was interrupted by the COVID-19 worldwide pandemic after three weeks of the six-week intervention. We decided to measure the impact of the intervention despite the pandemic interruption and also to use the qualitative ‘lived in’ experiences of the group to plan for the modified virtual intervention required in the post pandemic world.

### Methodology

This was a mixed methods feasibility study worked to that defined by Whitehead *et al.* (2014) to assess whether a full-scale study on the combined intervention of diet and exercise was possible in this patient group. Exploration of the patient’s experiences and perceptions during the initial COVID-19 pandemic was also included post pandemic with a view to redesigning the programme to run virtually.

### Participants

Thirty-two patients with knee osteoarthritis were originally recruited from the combined primary/secondary care waiting list. The inclusion criteria were a willingness to take part in the project, patients with knee osteoarthritis and a BMI greater than 27 kg/p/m^2^. Exclusion criteria were unwillingness to take part in the programme. Patients were recruited between September 2019 and January 2020 and the study commenced with the intervention group 16 patients on 27/02/20.

### Ethics

This feasibility study was approved by the Ethics committee of the North Eastern Health Board in September 2019, and it was modified to include a qualitative evaluation in September 2020.

### Intervention

Patients attended the ARMED to ESCAPE pain programme twice weekly supervised by Chartered Physiotherapists and were they also seen both individually and in a group session by a Chartered Dietitian. The ESCAPE-pain programme was delivered by ESCAPE-pain trained physiotherapists and the programme delivered was a structured education and exercise programme twice weekly as described on the website https://escape-pain.org/ESCAPE-pain. There was an additional module presented by a Nurse Specialist in Pain management on the ARMED to ESCAPE pain programme. The dietetic programme was designed by the presenting dietitian (RR) and was delivered in two phases.

### Phase 1

This was a 60-minute individual appointment with the dietitian for each participant in the dietetic department of Our Lady’s Hospital Navan. It involved an individual detailed nutrition and dietetic assessment. Participants were given an individualised meal plan, a guide to managing your weight booklet (Mullingar Weight Management booklet) and a health check form. Pre-assessment weight, waist circumference and BMI were recorded. Patients were scheduled also for three individual ‘*In person*’ review appointments and a final telephone review appointment at six months.

### Phase 2

This was a 60-minute group class for all participants. This class included explanation of the food pyramid and recommended servings. An interactive group session took place discussing the servings recommended and details re portion size. What to look for on the food label and how best to prepare food and health options were also discussed.

### Outcome assessments

During the first week of lockdown/third week of the intervention, the project was stood down by Slainte Care (DOH). On the re-introduction of the project in September 2020, all patients were contacted. Fifteen patients agreed to attend the hospital were then re-evaluated in the physiotherapy department. Outcome measurements included KOOS (Knee Injury and Osteoarthritis Outcome Score) (Roos *et al*. 1998), SWEMBS (Short Warwick-Edinburgh Mental Wellbeing Scale) (SWEMBS 2008), height, weight, waist circumference and BMI. Adherence to the programme as measured by attendance was also recorded.

On reassessment patients were also invited to take part in a semi-structured interview. An addendum for permission for this interview had been granted by the ethics committee prior to reassessment. The interviews were audio-recorded and followed a semi-structured interview approach. Each participant had a one-on-one semi-structured interview with the research assistant (KO’C), who is a female physiotherapist. Interviews lasted approximately 20 min and took place in physiotherapy department (see Appendix 2). The interviews aimed to explore the patients’ health and well-being generally over lockdown exploring particularly the effect of lockdown on their knee condition, weight management over lockdown and their appetite for virtual or online classes. The interview guide was developed by the principal researcher and carried out by the research assistant. Analysis was conducted with Prof Tara Cusack who has expertise in qualitative data collection and analysis

The interviews were transcribed verbatim by a commercial company and compared directly with the recordings by the research lead (BM). The lead researcher applied a thematic analysis with a semantic, mixed inductive and deductive approach to analyse the data, following the process outlined by Braun and Clarke [4]. After a data familiarisation stage, the data were categorised into codes. The relationships between codes were analysed, and themes were formed by grouping related codes together. The second researcher (TC) applied the same inductive thematic analysis process to review and code extracts from 13 transcripts. The two researchers compared codes and themes and resolved any differences in coding through discussion. Data saturation was determined when no new themes and relationships were found in the interview data. Final themes were agreed, defined and named by the two researchers. Illustrative quotes for each code and theme were identified.

## Results

Fifteen patients were reassessed. The average attendance was 92% with 6 patients attending all sessions, 5 attending 5/6, 1 attending 4/6 and 2 attending 3/6. One subject dropped out and 15/16 patients completed all outcome measurements.

Of these re-evaluated nine were female and six were male. One patient refused to attend due to COVID-19 health fears. The mean age attending the class was 61.7 years with a standard deviation of 10.6 years. The mean BMI pre-intervention was 38.1 kg/m^2^ with a standard deviation of 7.2 kg/m^2^. All patients completed the KOOS knee score (Roos *et al.* 1998) and the Short Warwick-Edinburgh Mental Well Being Scale (SWEMBS 2008) to evaluate anxiety and depression. There was a statistically significant improvement in pain, activities of daily living, quality of life and mental health and well-being scores from time one to time 2 with an eta scores of .4, .5 and .29 indicating large effect sizes.

(See Appendix 2)

The mean weight, BMI and waist measurements were reduced also from time one to time 2 but this failed to reach significance.

### Qualitative themes

Four major themes emerged from the interviews.

1. Enablers and barriers to coping in lockdown for this patient group. 2. Benefits of the ARMED programme structure to increasing physical activity. 3. Benefit of the ARMED programme structure to weight management. 4. Enablers and barriers to redesigning the programme for online delivery.

### Enablers and barriers to coping in lockdown

Within this theme both positive and negative coping methods were identified. Increased activity and social engagement were identified by several patients as positive coping strategies and similarly less social contact and reduced activity were not as easy to cope with. Sub-themes of the physical and psychological effects of lockdown were also identified for example isolation, decreased overall levels of function and increased weight.

“I just kept myself busy. I have my plants and my flowers and all that type of stuff, My son lives beside us and he did everything for us, so we wanted for nothing. So, I was happy”.

“I have been kept busy, you see, with the shop, it has been – it was very busy, the early stages was very busy and then, this next stage, is not as busy but still, I am kept going, you know, so that’s it like, so…Bored, fed up. Under pressure from the missus…. feeling isolated. I presume if you’re on your own for long enough you won’t come out of the house”.

### Benefit of the ARMED intervention

Nearly all participants reported the benefit of the ARMED active intervention. The sub-themes identified were the benefit of being in a group, the benefit of supervised exercise that was individually focussed and the fact that the exercises were fun and functional were repeatedly described. Many described keeping up the increase in activity since the classes ceased.

“I think that the environment that it created coming to it helped”.

“While you were in the group it was still very individual, you know, and it was good, it was good fun”.

“The weeks that we came, I was part of a group here; that was very beneficial to me. Even sometimes I think of what we were doing, and I think about what we were doing and then I’d sit down and start doing bits and pieces, you know”.

“I think the fact that they were controlled exercises and that the people I was with knew what they were doing, whereas I wouldn’t really know myself. I wouldn’t know what to do or how far to push it, or this or that, do you know that way?”

### Benefit of ARMED to enable weight loss

Overall, the weight management programme was also viewed as positive. Sub-themes explored were commitment to long-term changes, the advantages again of the group and the moderate, non-judgemental approach of the therapist.

… “And it is the only dietician you had there which I listened to because she was good. And it was grand - I never ate as much food in all my life after seeing that dietician because you were eating at the right times and you were eating the right stuff and it was a lot”.

“Sometimes if you are going one to one, you feel oh, God almighty, I am the only one, I am being singled out because of my weight or whatever, this course was good because just as a group thing it was good, and easier diet wise”.

### Enablers and barriers to telehealth

Many of those interviewed were positive about the possibility of attending a similar class online. Some, however, did not think the culture of the class would be preserved and felt given the age profile of participants technology literacy may prove problematic.

Two of the group would not be able to continue online “Well, we have no… Well like I have it on my phone, but I have no computer, like, you know”. “We don’t have the computer… We are not very well up on that”.

Most of the group identified positively with their ability to carry out the same class online. Again, the theme of belonging to a group and a sense of fun and connection were described. Even those without skills and technology at this time indicated they would find help to set it up. Some had extensive use of virtual classes and activities previously and welcome the initiative.

“Yes, my children would show me”.. “Ah yeah, well we have that - the meetings I go to, we are sort of like a family, we have developed a family thing”. “Yeah, I would love that…a kind of a Zoom thing? Yeah, oh, definitely, it would be nice to be gathered again where people would see the likes of you or the dietician….” “Yeah, probably would. You see, I have a laptop and I can put it onto the television, I have the cast, Chromecast that thing. We watch mass and all that on the television”.

## Discussion

The purpose of this study originally was to evaluate the feasibility of running an intervention (ARMED) to determine the effect of the combined diet and exercise programme in patients with knee osteoarthritis at the primary/secondary care interface. Although shorter due to the pandemic we found the combined programme was feasible with patients demonstrating good adherence and acceptability of the programme, reporting improvements in pain, increased overall activity, improved quality of life and mental health and well-being scores and reduced BMI with no adverse effects reported. Qualitatively, the patients were very positive about the combined programme. Only one patient failed to complete the follow-up. Indeed the results for this group intervention contrast with another pandemic study by Endstrasser (2020) of a similar patient group who reported increased pain and stiffness with reduced activity levels during the same lockdown period time, demonstrating some evidence to support the need for an intervention like this to preserve function in this patient group. Evaluation of preliminary data from this feasibility study supports the three-week intervention and would support the need for further evaluation of the total programme in more normal times.

The COVID-19 pandemic additionally offered us the opportunity to explore the patient’s experiences and perceptions of the programme qualitatively that we used to redesign the programme to run virtually when face-to-face programmes were not possible. Previous reviews by Cotteral *et al.* (2016) and Jiang (2018) had shown telehealth can provide improvements in pain and physical function for patients with knee pain but prior to COVID-19 this was not a widespread practice. Age, level of education and computer literacy had been previously cited as barriers to telehealth (Kruse *et al.* 2018). In contrast, this group reported high levels of satisfaction with the concept of a virtual health version of this programme with only a minority citing computer literacy as an issue. We think the patient’s perceptions of virtual health during/post this pandemic may have changed and the insight of these patients now are very valuable in the redesign of a virtual programme. A virtual programme may allow potentially earlier and effective intervention for primary care patients with knee osteoarthritis in their own homes post pandemic, but this will the effectiveness of this will require further evaluation.

Our study however has limitations. The absence of a control group due to the lockdown limits greatly the exploratory power of the results. This feasibility study has illustrated the complex nature of this combined intervention and has indicated the need to further evaluate the individual components, both to evaluate the activity of each ingredient alone and also to capture the value if any of the combined interventions. A future trial may require a three-arm evaluation Armed (Education, Diet and Exercise) Vs placebo, Escape (Education and exercise) Vs Placebo, Education and Diet intervention Vs Placebo. However, the positivity of the findings over the two time points does support the need for further evaluation of the ARMED intervention in the primary care knee osteoarthritic group.

Due to COVID-19 and the restrictions faced, we were no longer able to carry out a class like this in real time. The qualitative analysis, however, has allowed design of a virtual class. The group was in the main very open to attending a similar class online. Their other life experiences virtually over lockdown has improved their online skills. The qualitative analysis also highlighted the positive view of the novel ‘group’ approach by the dietitian in addition to the usual individual consults which will be included in the virtual redesign.

We have now redesigned our programme to present it virtually. This feasibility study gave us the evidence we needed to redesign the programme, and we hope to present the results of our virtual feasibility study in 2021. Indeed the provision of an evidence-based virtual programme may provide some solution to the findings of a recent review by Karasavvidis *et al.* (2020) who stated that home-based self-management strategies were necessary for the reduction in pain and maintenance of function in patients with knee osteoarthritis who have had their surgery postponed due to COVID-19.

## Conclusion

Evaluation of preliminary data from this feasibility study supports this intervention albeit a limited version combining education, exercise and weight management in this patient group even during a pandemic. It would support the need for further evaluation of the total programme in more normal times. Based on the results of the qualitative interviews, we have redesigned our programme to present it virtually. This pilot study gave us the evidence needed to redesign the programme, and we hope to present the results of our virtual feasibility study later in 2021.

## Appendix 1

**Table:**

Sample quotes	Sub themes	Main themes
“Bored. Fed up. Under pressure from the missus”. Feeling of isolation. I presume if you’re on your own long enough you won’t come out of the house. “When the lockdown came sort of everything went out the window and I didn’t even bother thinking about anything because all the focus was on listening to that and doing this and that”	Psychological effect of lockdown	Enablers and barriers to coping in lockdown
“Since March, well, I suppose because I have put on weight my knee has probably got a bit worse. Just a little bit too much additional eating and just missing work colleagues. I haven’t gone back to the workplace, so I miss that. Well, it has gone to hell, as I would say you can see by the weight I am there, that’s it. I am probably about 19 stone there roughly so… This year, I suppose, if this hadn’t have come along, I would definitely have been better”	Physical effect of lockdown	
“I would say I am 50% less active. As I say, I am working from home. I kind of come down and sit in the morning. I don’t get up until the afternoon and then get up for the evening and that’s - I really need to be aware. Whereas in the office I would have gone down to the canteen with the girls. Got up to go to the bathroom. Whereas I am only up to, because we have a bathroom downstairs, I don’t even have to take the stairs. I just kind of forget myself when I am sitting there, and next minute I go ‘God, I haven’t moved for about…’ Then I get up and I am crippled because I have sat for so long”.	Decreased function since lockdown	
“I just kept myself busy. I have my plants and my flowers and all that type of stuff. So, I was enjoying the time at home. I didn’t have to go anywhere for anything. My son lives beside us and he did everything for us so we wanted for nothing. So, I was happy”. “I have been kept busy, you see, with the shop, it has been – it was very busy, the early stages was very busy and then, this next stage, is not as busy but still, I am kept going, you know, so that’s it like, so… Okay, okay. I am fairly involved with the Men’s Shed; I think I told you that. I slip down there for a few hours in the mornings, to stay active, you know that way”.	Positive coping methods with lockdown	
“ I think that the environment that it created coming to it helped. While you were in the group it was still very individual, you know, and it was good, it was good fun. Yeah, yeah it definitely was helpful. The weeks that we came, I was part of a group here; that was very beneficial to me. Even sometimes I think of what we were doing, and I think about what we are doing and then I’d sit down and start doing bits and pieces, you know. Or and then I walk a good bit. it was improving with the movement. That really helped. And I don’t know, but I would imagine that would have had something to do with the swelling going down, you know. I don’t know. But I do, even if I am sitting now, I swing my legs in and out like that a lot”.	Physical group environment	Benefit of ARMED to increase activity
“I think the fact that they were controlled exercises and that the people I was with knew what they were doing, whereas I wouldn’t really know myself. I wouldn’t know what to do or how far to push it, or this or that, do you know that way?”	Supervised	
“Like we had a bit of fun as well, you know, it wasn’t just… Like you should know yourself; you were there, and the other people were all so lovely. I was really lucky with the crowd that was there. They were all friendly, you know. It was really nice”.	Fun element	
“I just liked the exercises. I liked the way they increased in time. The way they were simplified, a couple of stairs. You know, things you could do just on your own bed. And you know, it wasn’t forced on you. Even if I am just sitting, and what I do now - during the ads and that, now I don’t look at the telly much, only in the evening. It would be about seven before I would sit down to it. And during the ads I would get up and walk around because if I sit for too long, I go stiff, I don’t be able to stand up, you know. I wouldn’t be able to hold myself and that does good, you know”.	Functional	
“And it is the only dietician you had there which I listened to because she was good. And it was grand - I never ate as much food in all my life after seeing that dietician because you were eating at the right times and you were eating the right stuff and it was a lot”.	Advice for long term management	ARMED to enable weight loss S
“Excellent. Actually, I will pull that book out again because I thought it was the most simplified. We all agreed that that was the most simplified diet that we’d… Any of us had ever encountered. Because the first time that somebody said ‘Well, you can actually eat everything, but it is called everything in moderation’. And you know, so, I thought that was very useful so I must pull that out again. I enjoyed that and it worked. It was working without feeling you were on a diet”.	Simple approach Moderate approach	
“Sometimes if you are going one to one, you feel oh, God almighty, I am the only one, I am being singled out because of my weight or whatever. So, it was good and as I say it was good to compare that you are not really the only one and people are putting up with a lot of pain”.	Non judgemental	
“this course was good because just as a group thing it was good, and easier to - diet wise and things, it was easy to get it into your head and just do it”.	Group set up	
“Yeah, well the live class would be better than an actual video”.	Live class	Enablers and barriers to Telehealth
“Yeah, probably would. You see, I have a laptop and I can put it onto the television, I have the cast, Chromecast that thing. We watch mass and all that on the television”.	Computer literate	
“Yeah, I would love that…a kind of a Zoom thing? Yeah, oh, definitely, it would be nice to be gathered again where people would see the likes of you or the dietician” ….	Familiar	
“Well it’d save you from travelling”.	Cost effective	
“But I would, I’d be interested especially if they were live or if I was able to contact with” …	Live class	
“Well, we have no… Well like I have it on my phone, but I have no computer, like, you know. Well, I don’t have the things. We don’t have the computer or that, you know, we don’t… We are not very well up on that”.	No Computer/Barrier	
“are an older age group, right? So, I don’t think, you mightn’t, you won’t get the same interaction that you had there, people were starting to get to know each other there and were talking in the groups and things like that there, to do it electronically is, I don’t know….”	Older age? NOT as effective	
“Ah yeah, well we have that - the meetings I go to, we are sort of like a family, we have developed a family thing. And there would be the slagging for a half an hour. Then there would be the regular meeting which is serious stuff and then you would be slagging for a half an hour after it. So, it is a lively bit of craic. Then there would be people then from America, Germany, Iceland and different countries, come to the Irish one. They seem to like the Irish ones because there is a bit of craic towards - and there is no old dry stuff on it. I know some of the lads on it for forty years. So, we would know a lot of personal stuff and we would be slagging each other [laughs]. There is a fellow on it, Jack the Hand. He worked in O’Neill’s and he got his hand caught in the, you know, the thing for cutting the salt, the knife thing. It cut off his fingers and they nicknamed him Jack the Hand. That is the type of thing that goes on. It is brutal. If you have any deformity at all [laughs] they see it and they would slag you. It is a black sort of humour but sure, it is what it is”.	Strong sense of community with Zoom previously	
“Well I’m not online but I’d workout something”	Positive	
“Would you have the technology to do that? Erm… Yes, my children would show me”.	Positive	
